# Lunapark deficiency leads to an autosomal recessive neurodevelopmental phenotype with a degenerative course, epilepsy and distinct brain anomalies

**DOI:** 10.1093/braincomms/fcad222

**Published:** 2023-08-17

**Authors:** Andrea Accogli, Maha S Zaki, Mohammed Al-Owain, Mansour Y Otaif, Adam Jackson, Emanuela Argilli, Kate E Chandler, Christian G E L De Goede, Tülün Cora, Javeria Raza Alvi, Atieh Eslahi, Mahsa Sadat Asl Mohajeri, Setareh Ashtiani, P Y Billie Au, Alicia Scocchia, Kirsi Alakurtti, Alistair T Pagnamenta, Mehran Beiraghi Toosi, Ehsan Ghayoor Karimiani, Majid Mojarrad, Fatemeh Arab, Fahrettin Duymuş, Morris H Scantlebury, Gözde Yeşil, Jill Anne Rosenfeld, Ayberk Türkyılmaz, Safiye Güneş Sağer, Tipu Sultan, Farah Ashrafzadeh, Tatheer Zahra, Fatima Rahman, Shazia Maqbool, Mohamed S Abdel-Hamid, Mahmoud Y Issa, Stephanie Efthymiou, Peter Bauer, Giovanni Zifarelli, Vincenzo Salpietro, Zuhair Al-Hassnan, Siddharth Banka, Elliot H Sherr, Joseph G Gleeson, Pasquale Striano, Henry Houlden, Mariasavina Severino, Reza Maroofian

**Affiliations:** Division of Medical Genetics, Department of Specialized Medicine, McGill University, Montreal H3G 1A4, Canada; Department of Human Genetics, McGill University, Montreal, QC H3A 0C7, Canada; Clinical Genetics Department, Human Genetics and Genome Research Institute, National Research Centre, Cairo 12622, Egypt; Department of Medical Genomics, Center for Genomics Medicine, King Faisal Specialist Hospital and Research Center, Riyadh 11211, Saudi Arabia; Department of Pediatric, Neurology Section, Abha Maternity and Childern Hospital, Abha 62521, Saudi Arabia; Division of Evolution, Infection and Genomics, School of Biological Sciences, Faculty of Biology, Medicine and Health, University of Manchester, Manchester M13 9PT, UK; Manchester Centre for Genomic Medicine, University of Manchester, St Mary’s Hospital, Manchester Academic Health Science Centre, Manchester M13 9WL, UK; Department of Neurology, University of California, San Francisco, San Francisco, CA 94143, USA; Division of Evolution, Infection and Genomics, School of Biological Sciences, Faculty of Biology, Medicine and Health, University of Manchester, Manchester M13 9PT, UK; Manchester Centre for Genomic Medicine, University of Manchester, St Mary’s Hospital, Manchester Academic Health Science Centre, Manchester M13 9WL, UK; Department of Paediatric Neurology, Clinical Research Facility, Lancashire Teaching Hospital NHS Trust, Preston PR2 9HT, UK; Department of Medical Genetics, Selcuk University School of Medicine, Konya 42100, Turkey; Department of Pediatric Neurology, Institute of Child Health, Children's Hospital, Lahore 54590, Pakistan; Department of Medical Genetics, Faculty of Medicine, Mashhad University of Medical Sciences, Mashhad 917794-8564, Iran; Student Research Committee, Faculty of Medicine, Mashhad University of Medical Sciences, Mashhad 9137-86177, Iran; Department of Medical Genetics, Faculty of Medicine, Mashhad University of Medical Sciences, Mashhad 917794-8564, Iran; Alberta Children’s Hospital Research Institute, Department of Medical Genetics, University of Calgary, Alberta T2N 4Z6, Canada; Alberta Children’s Hospital Research Institute, Department of Medical Genetics, University of Calgary, Alberta T2N 4Z6, Canada; Blueprint Genetics Inc, Marlborough, MA 01752, USA; Blueprint Genetics Inc, Marlborough, MA 01752, USA; NIHR Biomedical Research Centre, Wellcome Centre for Human Genetics, University of Oxford, Oxford OX3 7BN, UK; Pediatric Neurology Department, Mashhad University of Medical Sciences, Mashhad 913791-6847, Iran; Neuroscience Research Center, Mashhad University of Medical Sciences, Mashhad 91375-33116, Iran; Molecular and Clinical Sciences Institute, St. George’s, University of London, Cranmer Terrace, London SW17 0RE, UK; Department of Medical Genetics, Next Generation Genetic Polyclinic, Mashhad 91869-51591, Iran; Department of Medical Genetics, Faculty of Medicine, Mashhad University of Medical Sciences, Mashhad 917794-8564, Iran; Student Research Committee, Faculty of Medicine, Mashhad University of Medical Sciences, Mashhad 9137-86177, Iran; Genetic Center of Khorasan Razavi, Mashhad 91877-53831, Iran; Department of Medical Genetics, Faculty of Medicine, Tehran University of Medical Sciences, Tehran 1411713135, Iran; Department of Medical Genetics, Selcuk University School of Medicine, Konya 42100, Turkey; Department of Medical Genetics, Konya City Hospital, Konya 42020, Turkey; Departments of Pediatrics and Clinical Neuroscience, University of Calgary; Alberta Children’s Hospital Research Institute, Hotchkiss Brain Institute & Owerko Center, University of Calgary, Alberta T2N 4N1, Canada; Department of Medical Genetics, Istanbul Medical Faculty, Istanbul University, Istanbul 34093, Turkey; Department of Molecular and Human Genetics, Baylor College of Medicine, Houston, TX 77030, USA; Baylor Genetics Laboratories, Houston, TX 77021, USA; Department of Medical Genetics, Karadeniz Technical University Faculty of Medicine, Trabzon 61080, Turkey; Clinics of Pediatric Neurology, Kartal Dr. Lütfi Kırdar City Hospital, İstanbul 34890, Turkey; Department of Pediatric Neurology, Institute of Child Health, Children's Hospital, Lahore 54590, Pakistan; Pediatric Neurology Department, Mashhad University of Medical Sciences, Mashhad 913791-6847, Iran; Department of Developmental-Behavioral Pediatrics, University of Child Health Sciences, The Children’s Hospital, Lahore 54590, Pakistan; Department of Developmental-Behavioral Pediatrics, University of Child Health Sciences, The Children’s Hospital, Lahore 54590, Pakistan; Department of Developmental-Behavioral Pediatrics, University of Child Health Sciences, The Children’s Hospital, Lahore 54590, Pakistan; Medical Molecular Genetics Department, Human Genetics and Genome Research Institute, National Research Centre, Cairo 12622, Egypt; Clinical Genetics Department, Human Genetics and Genome Research Institute, National Research Centre, Cairo 12622, Egypt; Department of Neuromuscular Diseases, UCL Queen Square Institute of Neurology, Queen Square, London WC1N 3BG, UK; CENTOGENE, Rostock 18057, Germany; CENTOGENE, Rostock 18057, Germany; Department of Neuromuscular Diseases, UCL Queen Square Institute of Neurology, Queen Square, London WC1N 3BG, UK; Department of Biotechnological and Applied Clinical Sciences, University of L’Aquila, L’Aquila 67100, Italy; Department of Medical Genomics, Center for Genomics Medicine, King Faisal Specialist Hospital and Research Center, Riyadh 11211, Saudi Arabia; College of Medicine, Alfaisal University, Riyadh 11533, Saudi Arabia; Division of Evolution, Infection and Genomics, School of Biological Sciences, Faculty of Biology, Medicine and Health, University of Manchester, Manchester M13 9PT, UK; Manchester Centre for Genomic Medicine, University of Manchester, St Mary’s Hospital, Manchester Academic Health Science Centre, Manchester M13 9WL, UK; Department of Neurology, University of California, San Francisco, San Francisco, CA 94143, USA; Department of Neurosciences, University of California, San Diego, La Jolla 92093, USA; Rady Children’s Institute for Genomic Medicine, San Diego 92123, USA; Department of Neurosciences Rehabilitation, Ophthalmology, Genetics, Maternal and Child Health (DiNOGMI), University of Genoa, Genoa 16132, Italy; Pediatric Neurology and Muscular Diseases Unit, IRCCS Istituto ‘Giannina Gaslini’, Genoa 16147, Italy; Department of Neuromuscular Diseases, UCL Queen Square Institute of Neurology, Queen Square, London WC1N 3BG, UK; Neuroradiology Unit, IRCCS Istituto Giannina Gaslini, Genoa 16146, Italy; Department of Neuromuscular Diseases, UCL Queen Square Institute of Neurology, Queen Square, London WC1N 3BG, UK

**Keywords:** endoplasmic reticulum, LNPK, ear-of-the-lynx sign, substantia nigra, corpus callosum hypoplasia

## Abstract

*LNPK* encodes a conserved membrane protein that stabilizes the junctions of the tubular endoplasmic reticulum network playing crucial roles in diverse biological functions. Recently, homozygous variants in *LNPK* were shown to cause a neurodevelopmental disorder (OMIM#618090) in four patients displaying developmental delay, epilepsy and nonspecific brain malformations including corpus callosum hypoplasia and variable impairment of cerebellum. We sought to delineate the molecular and phenotypic spectrum of *LNPK*-related disorder. Exome or genome sequencing was carried out in 11 families. Thorough clinical and neuroradiological evaluation was performed for all the affected individuals, including review of previously reported patients. We identified 12 distinct homozygous loss-of-function variants in 16 individuals presenting with moderate to profound developmental delay, cognitive impairment, regression, refractory epilepsy and a recognizable neuroimaging pattern consisting of corpus callosum hypoplasia and signal alterations of the forceps minor (‘ear-of-the-lynx’ sign), variably associated with substantia nigra signal alterations, mild brain atrophy, short midbrain and cerebellar hypoplasia/atrophy. In summary, we define the core phenotype of *LNPK*-related disorder and expand the list of neurological disorders presenting with the ‘ear-of-the-lynx’ sign suggesting a possible common underlying mechanism related to endoplasmic reticulum-phagy dysfunction.

## Teaser

In this study, Accogli *et al*. delineate the molecular and clinical spectrum of LNPK-related neurodevelopmental disorder with degenerative course, characterized by a recognizable neuroimaging pattern consisting of corpus callosum hypoplasia and signal alterations of the forceps minor (‘ear-of-the-lynx’ sign), suggesting a pathomechanism related to endoplasmic reticulum-phagy dysfunction.

## Graphical Abstract

**Graphical Abstract:**
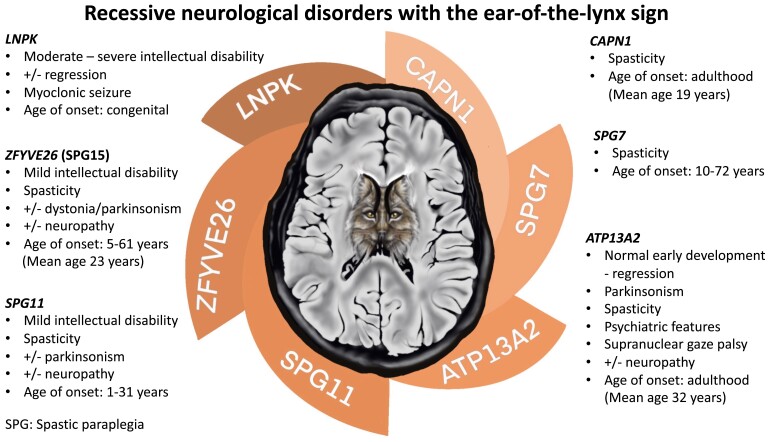

## Introduction

The endoplasmic reticulum (ER) is involved in diverse biological functions, including protein synthesis, folding and transport, carbohydrate metabolism, lipid and steroid synthesis and calcium homeostasis.^1,2^ The progressive understanding of ER structure and function in recent years has unravelled the role of ER dysfunction in several neurodegenerative disorders in humans, such as hereditary spastic paraplegia (SPG)^3^ and Parkinson disease.^4^

Lunapark (Lnp) is a conserved membrane protein that localizes preferentially to the three-way junctions connecting the tubular ER network.^5,6^ Through its ubiquitin ligase activity, it ubiquitinates atlastin-2 for the tubular network formation and stabilization of the junctions.^7,8^ In higher eukaryotes, phosphorylation of Lnp may contribute to the conversion of the ER from tubules to sheets during mitosis.^8^

Recently, three homozygous loss-of-function (LoF) variants in *LNPK* were shown to cause a neurodevelopmental disorder (OMIM#618090) in four patients displaying global developmental delay (GDD)/intellectual disability (ID), epilepsy, corpus callosum hypoplasia and variable impairment of cerebellar development.^9,10^

Here, we present 16 new individuals from 12 different families harbouring 11 novel homozygous LoF variants in *LNPK*, outlining the molecular and phenotypic spectrum of *LNPK*-related disorder.

## Materials and methods

### Patients and genetic analysis

**Figure 1:**
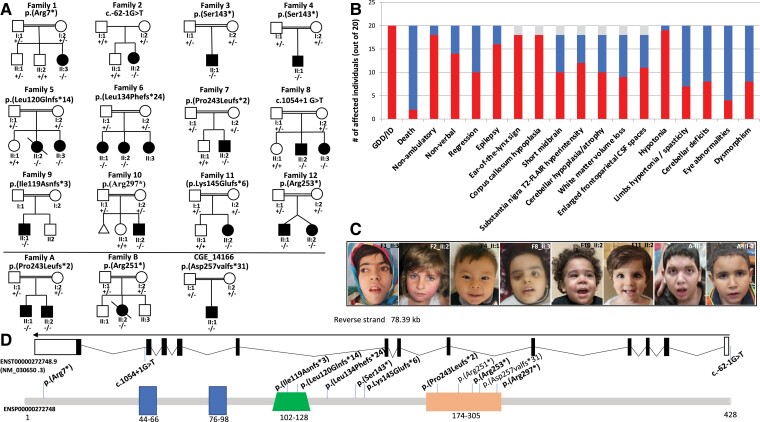
**Pedigrees of the families, photos, clinical summary of the affected individuals and schematic representation of the gene and protein with all the pathogenic variants.** (**A**) Pedigrees of Families 1–11. In the pedigrees, squares represent males, circles females and black shaded symbols denote patients harbouring biallelic *LNPK* variants. Plus (+) and minus (−) signs indicate absence or the presence of the *LNPK* variants [(+/+) wild-type, (+/−) heterozygote and (−/−) homozygote for the *LNPK* variant]. Pedigrees of previously reported patients are at the bottom of **A** separated by a line. (**B**) Bar graph showing the distribution of the most relevant clinical and radiological features among the total patients (20) identified so far with biallelic *LNPK* variants. Red: number of patients out of 18 showing each feature. Blue: number of patients without each specific feature. Grey: brain MRI not available for two individuals. GDD, global developmental delay; ID, intellectual disability (**C**) Clinical features of patients with homozygous *LNPK* variants showing subtle and nonspecific dysmorphic features such as medially flared eyebrows, long palpebral fissures, prominent philtrum, long chin in Patient II:3 of Family 1; bilateral infraorbital crease and thin upper lip vermilion in Patient II:2 of Family 2; almond-shaped eyes, anteverted nares and thin upper lip vermilion in Patient II:1 of Family 4; uplifted earlobes in Patient II:3 of Family 8; smooth philtrum, thin upper lip vermilion and uplifted earlobes in Patient II:2 of Family10; deep set eyes, thin upper lip vermilion and uplifted earlobes in Patient II:2 of Family 11; low frontal hairline and thick earlobes in Patients A-III-1 and A-III-2 previously published by Breuss *et al*.^9^ (**D**) Schematic depiction of transcript (ENST00000272748.9) and the full-length LNP protein (GenBank: NP_085153.1) showing two transmembrane domains (dark blue), a coiled coil region (green) and a zinc^2+^ finger domain (orange). The variants identified in the current cohort are displayed in bold. Note that the variant c.726del p.(Pro243Leufs*2) was also identified in the original manuscript of Breuss *et al*.^9^

Sixteen previously unreported patients from 12 families of different ancestries (Egyptian, Iranian, Turkish, Saudi Arabian, Afghan, Pakistani and British) as well as additional follow-up data from four patients reported from three families (Egyptian, Pakistani and Turkish)^9,10^ were included in this study after obtaining written informed consent (Fig. 1A).

Clinical data were collected using standardized pro forma from around 10 different hospitals and clinics, for all individuals. Brain magnetic resonance imaging (MRI) of these and of the previously reported patients^9,10^ were reviewed by an experienced neuroradiologist (M.S.). Exome sequencing (ES) or genome sequencing (Family 2) was performed in probands in the respective collaborating centres using slightly different analysis platforms according to the BWA/GATK-based pipelines. Sanger sequencing with standard methods was performed for candidate variants’ validation and familial segregation. All *LNPK* variants are reported according to the transcript NM_030650.3 and classified according to the American College of Medical Genetics and Genomics (ACMG) and the Association for Molecular Pathology (AMP) variant classification system. The study was approved by the University Colleague London (UCL) research ethics committee as well as institutional ethics committees of participating centres including medical research ethics of the National Research Centre (NRC) in Cairo, Egypt.

## Results

### Genetic findings

ES or genome sequencing revealed 12 novel or ultra-rare *LNPK* variants homozygous in affected individuals as follows: a nonsense variant c.19C>T p.(Arg7*) in Family 1, a splicing variant c.-62-1G>T in Family 2, a nonsense variant c.428C>A p.(Ser143*) in Families 3 and 4, a frameshift c.359_362del p.(Leu120Glnfs*14) in Family 5, a frameshift variant c.402_405del p.(Leu134Phefs*24) in Family 6, a frameshift variant c.726del p.(Pro243Leufs*2) in Family 7, the splicing variant c.1054+1G>T in Family 8, a frameshift variant c.355dup p.(Ile119Asnfs*3) in Family 9, a nonsense variant c.889C>T p.(Arg297*) in Family 10, a frameshift variant c.431dup p.(Lys145Glufs*6) in Family 11 and a nonsense variant c.757C>T p.(Arg253*) in Family 12 (Fig. 1A–D). Ten of these variants were novel, while the c.726del p.(Pro243Leufs*2) was previously reported in an unrelated family from Egypt.^9^

For Family 2, homozygosity was due to uniparental isodisomy involving the entire Chromosome 2, and only the mother was a heterozygous carrier. Sanger sequencing confirmed segregation of the variants with the phenotype within these families.

All variants were classified as pathogenic according to the ACMG/AMP criteria and are extremely rare in human population variant databases (allele frequency ranging from 0 to 0.000003995 in gnomAD, UK Biobank and Queen Square genomics database). None of the variants were reported in a homozygous state in healthy individuals.

All nonsense and frameshift variants are predicted to result in a premature truncation of the transcript, likely leading to nonsense-mediated mRNA decay. The variants c.-62-1G>T and c.1054+1G>T are predicted to severely impair the protein structure through aberrant mRNA splicing (acceptor loss 0.98 score and donor loss 0.99 score, according to the SpliceAI tool).^11^ No other pathogenic/likely pathogenic variants were identified in the currently known neurodevelopmental or neurodegenerative disorder (NDD)–related genes.

### Clinical and neuroradiological findings

All 16 patients (9 females, 7 males; mean age 8.2, range 2–19) had GDD and moderate-to-profound ID (moderate = 5; severe = 8; profound = 3). Only one individual was able to walk with support at the last follow-up visit, and all were mostly nonverbal. Developmental regression was observed in seven, mostly occurring after seizure onset. One individual (II:2 of Family 5) died at the age of 9.5 years due to status epilepticus in the context of respiratory infection. Twelve individuals had epilepsy, experiencing different seizure types with a predominance of myoclonic and tonic–clonic seizures, and the age of onset was between 2 months and 6 years. For nine of them, epilepsy was refractory to antiseizure medications. Review of available EEG for 11 patients (including 2 previously reported) did not identify a specific electrographic pattern. Additional details about EEG findings are available in Supplemental Table 1 and other Supplementary material. Two patients were diagnosed with autism spectrum disorder while no major behavioural abnormalities were noted in other children. Neurological exam demonstrated axial hypotonia (*n* = 16), hyporeflexia (*n* = 6), limb hypertonia (*n* = 4), cerebellar tremor (*n* = 3) and ataxic gait in one of the two patients who were able to walk prior to regression. Ophthalmological findings included strabismus (*n* = 5), nystagmus (*n* = 4), bilateral cataracts (*n* = 2) and optic atrophy (*n* = 1). Two individuals had postnatal microcephaly, and another two showed mild macrocephaly, while the majority had normal head circumference. Subtle and nonspecific dysmorphic features were noticed in those individuals for whom photos were available (Fig. 1C).

**Figure 2:**
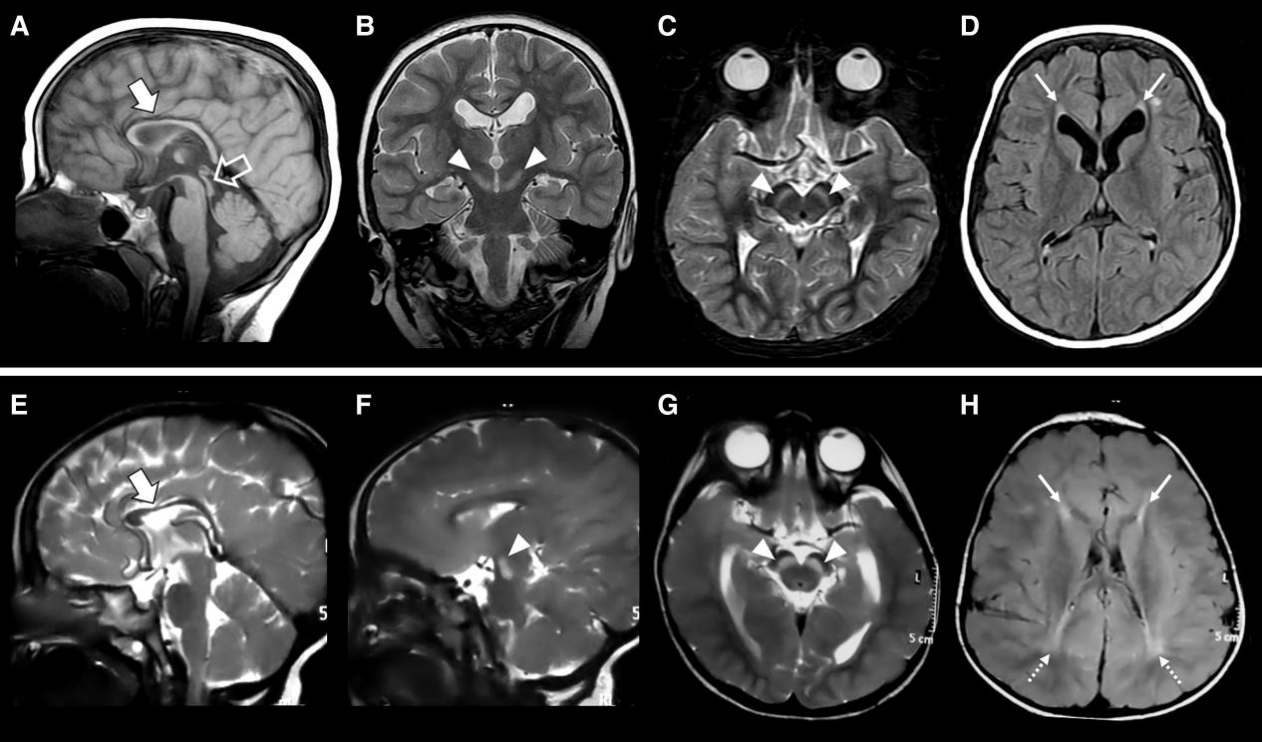
**Neuroimaging features of LNPK-related disorder.** Brain MRI studies performed in Patient II:1 from Family 6 at 4 years of age (**A–D**) and in Patient II:2 from Family 7 at 2.5 years of age (**E–H**). Sagittal T_1_- (**A**) or T_2_-weighted (**E**) images demonstrate corpus callosum hypoplasia with prevalent involvement of the anterior portions (thick arrows). Coronal (**B**), axial (**C, G**) and sagittal (**F**) T_2_-weighted images reveal symmetric marked T_2_ hyperintensity of the substantia nigra (arrowheads). Note the ‘ears-of-the-lynx’ sign (thin arrows) on axial FLAIR images (**D, H**) consisting of hyperintense signal of the forceps minor bilaterally, which resembles the shape of the ears of a lynx with their characteristic apical hair tuft. Additional posterior periventricular white matter signal alterations are noted in Patient II:2 from Family 7 (dotted arrows). A short midbrain is also visible in Patient II:1 from Family 6 (empty arrow).

Brain MRI studies were available for review in 18 patients (14 from the present cohort and 4 from previous publications^9,10^; mean age at MRI: 4.6 years, range 1–14 years) (Supplementary Fig. 1). In all patients (18/18, 100%), we found callosal hypoplasia with prevalent anterior involvement and focal signal changes of the forceps minor of the corpus callosum reminiscent of the ‘ear-of-the-lynx’ sign (Fig. 2). Additional prominent features included bilateral symmetric T_2_-Fluid attenuated inversion recovery (FLAIR) hyperintensity of the substantia nigra (13/18, 72.2%), enlargement of the cerebral CSF spaces (11/18, 61.1%), a short midbrain (10/18, 55.5%), white matter volume loss with an antero-posterior gradient (9/18, 50%), mild inferior vermis hypoplasia (8/18, 44.4%) and other periventricular white matter signal alterations (8/18, 44.4%). Mild cerebellar atrophy (3/18, 16.6%) was detected in a subset of patients (Supplementary Fig. 2). Clinical features are summarized in Table 1 and Fig. 1D and extensively available in Supplementary Table 1.

**Table 1:** Genetic and phenotypic characteristics of patients with *LNPK* variants

Family ID	1	2	3	4	5		6		7	8		9	10	11	12		13			14
Patient	II:3	II:2	II:1	II:1	II:2	II:3	II:1	II:2	II:2	II:2	II:3	II:1	II:2	II:2	II:1	II:2	A-III-1	A-III-2	B-III-2	CGE_14
Age, sex	13y, F	16y, F	13y, M	3y5m, M	9y, F died	2.5y, F	19y, F	15y, F	7y, M	7y, M	3y, F	12y, M	3y, M	2y, F	3y,M	3y,F	15y, M	7y4m, M	16y, F died	9y, F
GDD/ID	++++	+++	++++	+++	+++	++	+++	++	++++	+++	++	+++	++	++	+++	+++	+++	+++	+++	+++
Nonambulate	+	+ ^a^	+	+	+	+	+	+	+	+	+	+^a^	+	+	+	+	+	−	−	+^a^
Nonverbal	+	−	+	+	+	+	+	+	+	+	+	+	−	−	+	−	+	−	−	+
Regression	+	−	+	−	+	−	+	−	+	−	+	+	−	−	−	−	+	−	+	+
Epilepsy	+	+	+	+	+	−	+	+	+	+	+	+	−	−	+	−	+	+	+	+
Seizure-AOO	10m	6y	4y	2m	3y		2y	18m	2y	4y	2y	5y			2y3m		2y	2y	6y	7y
Seizure type	Myo, TC	Myo, TC	Myo	Focal, TC	Myo, TC		TC, atyp Abs.	NA	Myo	Focal TC	TC	Myo, TC			Myo, TC		Myo	Myo	TC	Myo, TC
Seizure frequency	Up to 100/day	3–4/week	4–5/day	1–2/month	20/day		20/day	NA	30–50/day	1/month	1–2/day	3–4/day			NA		Up to 10/day	NA	Up to 10/day	Up to 20/day
Response to ASM	−	+	−	+	−		−	NA	−	−	−	−			−		−	+	−	−
Age at brain MRI	8y3m	7y	8y	1y	1y1m	NA	4y10m	1y6m	2y5m	3y	4y	8m; 1y9m	3y	2y	NA	2y	6y	4y	14y	2y7m; 9y
CCH	+	+	+	+	+	NA	+	+	+	+	+	+	+	+	NA	+	+	+	+	+
Ears of lynx sign	+	+	+	+	+	NA	+	+	+	+	+	+	+	+	NA	+	+	+	+	+
WMVL	−	−	+++	−	−	NA	++	++	−	++	++	−	−	−	NA	−	+	+	+	++
Enlarged FP CSF spaces	+	−	+	+	+	NA	+	+	−	+	+	+	−	−	NA	−	−	−	+	+
Midbrain height	Short	Short	Normal	Short	Normal	NA	Short	Short	Normal	Short	Short	Normal	Normal	Short	NA	Normal	Normal	Normal	Short	Short
Substantia nigra SA	−	+	+	−	−	NA	+	+	+	+	+	−	+	+	NA	+	−	−	+	+
Cerebellum	Mild atrophy	Normal	Mild atrophy, IVH	Normal	Mild IVH	NA	Mild IVH	Mild IVH	Mild IVH	Normal	Normal	Mild IVH	Normal	Normal	NA	Normal	Normal	Mild IVH	Mild atrophy	Mild IVH
OFC (SDS)	−0.9	−3.2	+0.5	−1.2	+0.6	+0.2	+3.3	+2.5	−2.6	+1.1	+1	−1.1	−0.1	−2.4	NA	NA	−1.1	−1.0	−1.4	+1.14
Axial hypotonia	+	+	+	+	+	+	+	+	+	+	+	+	+	+	+	+	+	+	+	−
Limbs hypertonia	−	+	−	−	+	−	−	−	−	+	+	+	−	−	−	−	+	+	−	+
Cerebellar signs	−	+	−	−	−	−	−	−	+	−	−	+	−	−	−	−	−	+	−	+
Dysmorphism	+	+	+	+	−	−	−	−	−	+	+	+	+	−	−	−	−	−	−	−
Eye features	−	Bil. cataracts	Nystagmus	Bil. ONA	Nystagmus esotropia	Esotropia	−	Esotropia	Nystagmus	−	−	Esotropia, Bil. cataracts	−	−	−	−	−	−	Bil. ONA	−

+ and − denote the presence or absence of a specific feature, respectively. Families 13 and 14 have been reported by Breuss *et al*.^9^ and Türkyılmaz *et al*.,^10^ respectively. ASM, antiseizure medications; AOO, of onset; atyp, atypical; Bil., bilateral; CCH, corpus callosum hypoplasia; GDD, global developmental delay; DTR, deep tendon reflexes; F, female; FP, frontoparietal; hom, homozygous; myo, myoclonic; TC, tonic–clonic; ID, intellectual disability; IVH, inferior vermis hypoplasia; m, months; OFC, occipital frontal circumference; ONA, optic nerve atrophy; M, male; NA, not available; SA, signal alterations; SDS, standard deviations; WMVL, white matter volume loss; y, years.

a Previously able to walk; unable to walk after regression.

## Discussion

All affected individuals of our and previous cohorts^9,10^ harbour LoF homozygous variants in *LNPK,* resulting in a neurodevelopmental phenotype characterized by moderate to profound DD/ID, refractory epilepsy and a recognizable neuroradiological pattern. Interestingly, brain MRI analysis including review of previously published patients led us to identify a consistent neuroimaging phenotype characterized by callosal hypoplasia and abnormal signal of the forceps minor (‘ear-of-the-lynx’ sign), variably associated with substantia nigra signal alterations, mild brain atrophy, short midbrain and cerebellar hypoplasia/atrophy. Of note, the ‘ear-of-the-lynx’ sign has been typically described in SPG11 (MIM#604360) and SPG15 (MIM#270700),^12^ linked to pathogenic variants in genes encoding spatacsin (*SPG11*) and spastizin (*ZFYVE26*), respectively, which play pivotal roles in intracellular trafficking and are part of a multiprotein complex important for ER function.^13-15^ The presence of this sign in the LNPK-related disorder further underscores the importance of ER for axon development and function.^3^ Moreover, signal alterations of the forceps minor with an ‘ear-of-the-lynx’ or ‘ear-of-the-grizzly’ morphology have been recently described in AP-4-associated hereditary SPG (AP-4-SPG)^16^ and in the allelic disorders SPG78 (MIM#617225) and Kufor–Rakeb syndrome (MIM#606693), due to biallelic variants in *ATP13A2*.^17^ The ‘ear-of-the-lynx’ sign has been also occasionally reported in patients with variants in the *SPG7* and *CAPN1* genes, linked to SPG7 (MIM#607259) and SPG76 (MIM #616907), respectively.^18,19^ Notably, several genes associated with the ‘ear-of-the-lynx’ sign such as *SPG11*,^15^*ZFYVE26*,^15^*ATP13A2*^17^ and *LNPK*^20^ have been implicated in autophagy, raising the suspicion for a possible common underlying mechanism related to ER-phagy dysfunction. Interestingly, myoclonic seizure is frequently observed in our cohort while it does not typically occur in the above disorders. This association when present may help clinicians to recognize LNPK-related disorder in the clinical setting. Main features of the NDD disorders presenting with the ‘ear-of-the-lynx’ sign and comparison with LNPK are displayed in the Supplementary Table 2.

In addition, most patients (72.2%) had additional T_2_-FLAIR hyperintensity of the substantia nigra. Remarkably, loss of normal susceptibility signal dropout of the substantia nigra is found in some neurodegenerative disorders such as Parkinson disease and related conditions^21^ in which the nigrostriatal pathway is impaired. However, signal alterations of the substantia nigra are unusual in neurodevelopmental disorders and have never been described in the group of SPGs linked to ER protein dysfunction. Notably, *LNPK* is abundantly expressed in the human substantia nigra (normalized protein-coding transcripts per million: 9.2 according to the Human Protein Atlas database), yet its role in the nigrostriatal dopaminergic circuit remains to be investigated. Neurological follow-up of affected individuals with *LNPK* pathogenic variants will be important to determine whether they may develop parkinsonism later in life like in the *ATP13A2*-related disorders, which could be potentially treated.

The effect of LNP deficiency on ER has previously been elucidated by knockout studies in *Saccharomyces cerevisiae*^6^ and mammalian cell lines,^8^ showing that its loss leads to a reduction of tubules and junctions and an increased sheet-like appearance at the cellular periphery, overall affecting the abundance of the three-way junctions. In humans, fibroblasts of patients harbouring a homozygous truncating variant in *LNPK* exhibited aberrant ER shape and increased luminal mass density.^9^ Likewise, we expect that the homozygous LoF variants identified in our patients result in a loss of protein function with consequent perturbation of ER morphology and homeostasis. However, the mechanism underlying impact on central nervous system development, resulting in cognitive impairment, epilepsy and brain malformations, is yet to be elucidated. The typical biphasic disease course with a neurodegenerative phase occurring on the background of a neurodevelopmental impairment may support at least in part a pathomechanism related to autophagy dysfunction as seen in other congenital disorders of autophagy.^22^ Of note, autophagosomes form at the ER in mammals, and ER membrane contacts are known to play a central role in regulating autophagosome formation.^23^ Although we may speculate that LNP deficiency impairs ER homeostasis and function with consequent perturbation of autophagy, a direct functional linkage between LNP and autophagosomes remains elusive and related signalling pathways yet unknown.

Furthermore, it is unknown why spasticity is not a major finding in individuals with LNP deficiency in contrast to the SPG phenotype of individuals with pathogenic variants in other ER genes. Finally, deletion of the *LNPK* homologue (lnp-1) in *Caenorhabditis elegans* causes mislocalization of presynaptic proteins, suggesting a role of Lnp-1 in synaptogenesis through regulation of vesicular transport or localization.^24^ This finding is in line with the clinical presentation of refractory epilepsy in our cohort, pointing to a possible synaptic dysfunction due to LNP deficiency.

In summary, we outline the clinical features of the LNPK-related NDD, mainly characterized by moderate to profound ID, epilepsy and recognizable brain anomalies. Specifically, the ‘ear-of-the-lynx’ sign associated with corpus callosum hypoplasia and substantia nigra signal alterations are the key feature that could guide clinicians toward an early clinical diagnosis. Further studies are needed to elucidate the LNP’s role in ER of developing neurons and the exact pathomechanism leading to LNP deficiency.

## Supplementary material

Supplementary material is available at *Brain Communications* online.

## Supplementary Material

fcad222_Supplementary_Data

## Data Availability

All variants have been deposited into the LOVD database: https://databases.lovd.nl/shared/variants/KIAA1715/unique.
