# Supplementary material for: Lunapark deficiency leads to an autosomal recessive neurodevelopmental phenotype with a degenerative course, epilepsy and distinct brain anomalies
Source: Brain Commun. 2023 Aug 17;5(5):fcad222. doi: 10.1093/braincomms/fcad222 (PMC10546953; doi:10.1093/braincomms/fcad222)
Supplement: fcad222_Supplementary_Data [file fcad222_supplementary_data.zip › Supplementary_table_1.docx]

**Supplemental Table 1**. **Detailed genetic and phenotypic characteristics of patients with *LNPK* variants.**

| **Family ID** | **Family 1** | **Family 2** | **Family 3** | **Family 4** | **Family 5** | | **Family 6** | | **Family 7** | **Family 8** | | **Family 9** | **Family 10** | **Family 11** | **Family 12** | | **Breuss et al. 2018** | | | **Türkyılmaz et al. 2022** |
| --- | --- | --- | --- | --- | --- | --- | --- | --- | --- | --- | --- | --- | --- | --- | --- | --- | --- | --- | --- | --- |
| **Subject** | II:3 | II:2 | II:1 | II:1 | II:2 | II:3 | II:1 | II:2 | II:2 | II:2 | II:3 | II:1 | II:2 | II:2 | II:1 | II:2 | A-III-1 | A-III-2 | B-III-2 | CGE_14166 |
| ***LNPK* variant (NM_030650.3**  **ENST00000272748.9)** | c.19C>T p.(Arg7*) | c.-62-1G>T | c.428C>A p.(Ser143*) | c.428C>A p.(Ser143*) | c.359_362del p.(Leu120Glnfs*14) | | c.402_405del p.(Leu134Phefs*24) | | c.726del p.(Pro243Leufs*2) | c.1054+1G>C | | c.355dup p.(Ile119Asnfs*3) | c.889C>T p.(Arg297*) | c.431dup  (p.Lys145Glufs*6) | c c.757C>T (p.Arg253*) | | c.726del, p.(Pro243Leufs*2) | | c.751C>T, p.(Arg251*) | c.770del, p.(Asp257valfs*31) |
| **Zygosity** | hom | hom | hom | hom | hom | | hom | | hom | hom | | hom | hom | hom | hom | | hom | hom | hom | hom |
| **Ethnicity** | Iranian | British | Iranian | Afghan | Egyptian | | Saudi Arabian | | Egyptian | Saudi Arabia | | Turkish | Egyptian | Pakistan | Arab | | Egyptian | Egyptian | Pakistan | Turkish |
| **Age, sex** | 13y, F | 16y, F | 13y, M | 3y5m, M | 9y, F | 2.5y, F | 19y, F | 15y, F | 7y, M | 7y, M | 3y, F | 12y, M | 3y, M | 2y, F | 3y,M | 3y,F | 15y, M | 7y4m, M | 16y, F | 9y, F |
| **Alive** | + | + | + | + | - | + | + | + | + | + | + | + | + | + | + | + | + | + | - | + |
| **GDD/ID** | Profound | Severe | Profound | Severe | Severe | Moderate | Severe | Moderate | Profound | Severe | Moderate | Severe | Moderate | Moderate | Severe | Severe | Severe | Severe | Severe | Severe |
| **Non-ambulatory** | + | + * | + | + | + | + | + | + | + | + | + | +* | + | + | + | + | + | - | - | +* |
| **Non-verbal** | + | - | + | + | + | + | + | + | + | + | + | + | - | - | + | - | + | - | - | + |
| **Regression** | + | - | + | - | + | - | + | - | + | - | + | + | - | - | - | - | + | - | + | + |
| **Epilepsy** | + | + | + | + | + | - | + | + | + | + | + | + | - | - | + | - | + | + | + | + |
| **Seizure-age of onset** | 10m | 6y | 4y | 2m | 3y |  | 2y | 18m | 2y | 4y | 2y | 5y |  |  | 2y3m |  | 2y | 2y | 6y | 7y |
| **Seizure type** | Myo, TC | Myo, TC | Myo | Focal, TC | Myo, TC |  | TC, atypical absences | NA | Myo | Focal TC | TC | Myo, TC |  |  | Myo, TC |  | Myo | Myo | TC | Myo, TC |
| **Seizure frequency** | Up to 100/day | 3-4/week | 4-5/day | 1-2/month | 20/day |  | 20/day | NA | 30-50/day | 1/month | 1-2/day | 3-4/day |  |  | NA |  | Up to 10/day | NA | Up to 10/day | Up to 20/day |
| **EEG findings** | Focal theta/delta bursts; slow wave-sharp waves | High amplitude rhythmic delta activity, frontal spikes and waves | Theta/delta bursts, slow wave-sharp waves | Bilateral spikes and slow waves, suppressed baseline activity | Slow-wave/sharp waves, modified hypsarrhythmia |  | NA | NA | Fast rhythms, disorganized background activity | Unremarkable | Unremarkable | High amplitude  multifocal spikes and spike-wave discharges |  |  | NA |  | NA | NA | Unremarkable | Unremarkable |
| **Response to ASM** | - | + | - | + | - |  | - | NA | - | - | - | - |  |  | - |  | - | + | - | - |
| **Age at brain MRI** | 8y3m | 7y | 8y | 1y | 1y1m | NA | 4y10m | 1y6m | 2y5m | 3y | 4y | 8m; 1y9m | 3y | 2y |  | 2y | 6y | 4y | 14y | 2y7m; 9y |
| **CCH** | + | + | + | + | + | NA | + | + | + | + | + | + | + | + | NA | + | + | + | + | + |
| **Ear-of-the-lynx sign** | + | + | + | + | + | NA | + | + | + | + | + | + | + | + | NA | + | + | + | + | + |
| **WMVL** | - | - | Severe | - | - | NA | Moderate | Moderate | - | Moderate | Moderate | - | - | - | NA | - | Mild | Mild | Mild | Moderate |
| **Enlarged FP CSF spaces** | + | - | + | + | + | NA | + | + | - | + | + | + | - | - | NA | - | - | - | + | + |
| **Midbrain height** | Short | Short | Normal | Short | Normal | NA | Short | Short | Normal | Short | Short | Normal | Normal | Short | NA | Normal | Normal | Normal | Short | Short |
| **Substantia nigra SA** | - | + | + | - | - | NA | + | + | + | + | + | - | + | + | NA | + | - | - | + | + |
| **Cerebellum** | Mild atrophy | Normal | Mild atrophy, IVH | Normal | Mild IVH | NA | Mild IVH | Mild IVH | Mild IVH | Normal | Normal | Mild IVH | Normal | Normal | NA | Normal | Normal | Mild IVH | Mild atrophy | Mild IVH |
| **OFC (SDS)** | -0.9 | -3.2 | +0.5 | -1.2 | +0.6 | +0.2 | +3.3 | +2.5 | -2.6 | +1.1 | +1 | -1.1 | -0.1 | -2.4 | NA | NA | -1.1 | -1.0 | -1.4 | +1.14 |
| **Neurological exam** | Hypotonia, ↓DTR | Axial hypotonia, limb hypertonia, ataxia | Hypotonia, ↓ DTR, nystagmus | Hypotonia, ↓ DTR, esotropia | Hypotonia, quadriparesis, nystagmus, esotropia | Hypotonia, nystagmus, esotropia | Hypotonia | Hypotonia, esotropia | Hypotonia, nystagmus, mild tremor | Hypotonia, ↓ DTR | Hypotonia, ↓DTR, limb hypertonia Babinski | Axial hypotonia, limb hypertonia, cerebellar tremor, esotropia | Hypotonia, ↓ DTR | Hypotonia | Hypotonia | Hypotonia | Axial hypotonia, spasticity and spasticity | Mild hypotonia, spasticity,wide based gait | Hypotonia | Limb hypertonia, hypereflexia, Babinski, ataxia |
| **Others** | - | Bilateral cataracts | - | Bilateral ONA | - | ASD | - | - | - | - | - | Bilateral cataracts, pectus excavatum | Mild autism | - | - | - | - | - | Bilateral ONA | - |
| **Dysmor-phism** | + | + | + | + | - | - | - | - | - | - | + | + | + | + | - | - | - | - | - | - |

ASD, Autism spectrum disorder; ASM, antiseizure medications; CCH, corpus callosum hypoplasia; CSF, cerebrospinal fluid; GDD, global developmental delay; DTR, deep tendon reflexes; F, female; FP, frontoparietal; hom, homozygous; myo, myoclonic; TC, tonic clonic; ID, intellectual disability; IVH, inferior vermis hypoplasia; m, months; OFC, occipital frontal circumference; ONA, optic nerve atrophy; M, male; NA, not available; SA, signal alterations; SDS, standard deviations; WMVL, white matter volume loss; y, years; * previously able to walk, unable to walk after regression; + and - present or absence of a specific feature respectively.
