# Supplementary material for: Lunapark deficiency leads to an autosomal recessive neurodevelopmental phenotype with a degenerative course, epilepsy and distinct brain anomalies
Source: Brain Commun. 2023 Aug 17;5(5):fcad222. doi: 10.1093/braincomms/fcad222 (PMC10546953; doi:10.1093/braincomms/fcad222)
Supplement: fcad222_Supplementary_Data [file fcad222_supplementary_data.zip › Supplementary_table_2docx.docx]

**Supplemental table 2. Main features and comparison of disorders presenting with the ear-of-the-lynx sign**

|  | **LNPK-related disorder** | **SPG11** | **SPG15** | **AP-4-SPG**** | **SPG78 / Kufor-Rakeb syndrome** | ***SPG7*** | **SPG76** |
| --- | --- | --- | --- | --- | --- | --- | --- |
| **Gene** | *LNPK* | *KIAA1840* | *ZFYVE26* | *AP4B1 AP4M1 AP4E1 AP4S1* | *ATP13A2* | *SPG7* | *CAPN1* |
| **Inheritance** | AR | AR | AR | AR | AR | AD,AR | AR |
| **Age of onset** | Congenital | 1-31 years | 5-61 years  (Mean age 23 years) | <1 year | Adulthood (Mean age 32 years) | 10-72 years | Adulthood (Mean age 19 years) |
| **DD/ID** | Moderate to profound | Mild | Mild | Severe | Usually normal early development | - | - |
| **Regression** | +/- | +/- cognitive decline | - | + | +/- cognitive decline/dementia* | - | - |
| **Epilepsy** | + (Myoclonic seizure ++) |  | Very rare | 50% (febrile seizure, focal/generalized motor seizure) | - | - | - |
| **Hypotonia** | + | - | - | + | - | - | - |
| **Spasticity** | - | + | + | + | + | + | + |
| **Extrapyramidal movements** | - | +/- Parkinsonism | +/- (dystonia, parkinsonism) | - | Parkinsonism* | - | - |
| **Cerebellar dysfunction** | +/- | +/- | +/- | +/. | + | +/- | +/- |
| **EOM defects** | +/- | +/- | +/- | +/.- | + SNP | +/- | +/- |
| **Sensory defects** | - | + neuropathy | +/- neuropathy | - | +/- neuropathy | +/. | +/- |
| **Ear-of-the-lynx sign** | + | + | + | +/- | +/- | +/- | +/- |
| **Thin CC** | + | + | + | + | + | +/- | +/- |
| **Other** | Substantia nigra SA | - | - | - | - | Dentate nuclei SA | - |
| **WM VL/SA** | +/- | +/- | +/- | + | +/- | +/- | +/- |
| **Short midbrain** | +/- | - | - | - | - | - | - |
| **Cerebellar atrophy** | +/- | +/- | +/- | +/- | +/- | +/- | +/- |
| **Cerebral cortex atrophy** | +/- | +/- | +/- | +/- | +/- | +/- | +/- |
| **Others** | +/- ONA, cataract | +/- Retinal degeneration, pes cavus scoliosis | +/- ONA, retinopathy, cataracts, SNHL, pes cavus scoliosis | Microcephaly, foot deformities, short stature stereotypic laughter w/tongue protrusion | Aggression, hallucination, mask-like facies, anosmia, psychotic* | +/- ONA, pale optic disk, hearing loss, pes cavus scoliosis | - |

*Parkinsonism, psychiatric features and cognitive decline are more pronounced in the in Kufor-Rakeb syndrome than in SPG78

**P-4-HSP: SPG47, SPG50, SPG51, SPG52

Legend: AD autosomal dominant, AR autosomal recessive, ASD autism spectrum disorder, CC corpus callosum, EOM extraocular movements, SA, signal alterations, VL volume loss, ONA optic nerve atrophy, SPG, SNP supranuclear gaze palsy, WM white matter.
