# Supplementary material for: Lunapark deficiency leads to an autosomal recessive neurodevelopmental phenotype with a degenerative course, epilepsy and distinct brain anomalies
Source: Brain Commun. 2023 Aug 17;5(5):fcad222. doi: 10.1093/braincomms/fcad222 (PMC10546953; doi:10.1093/braincomms/fcad222)
Supplement: fcad222_Supplementary_Data [file fcad222_supplementary_data.zip › EEG findings_supplementary data_PS.docx]

## Supplementary data. EEG findings in the LNPK patients.


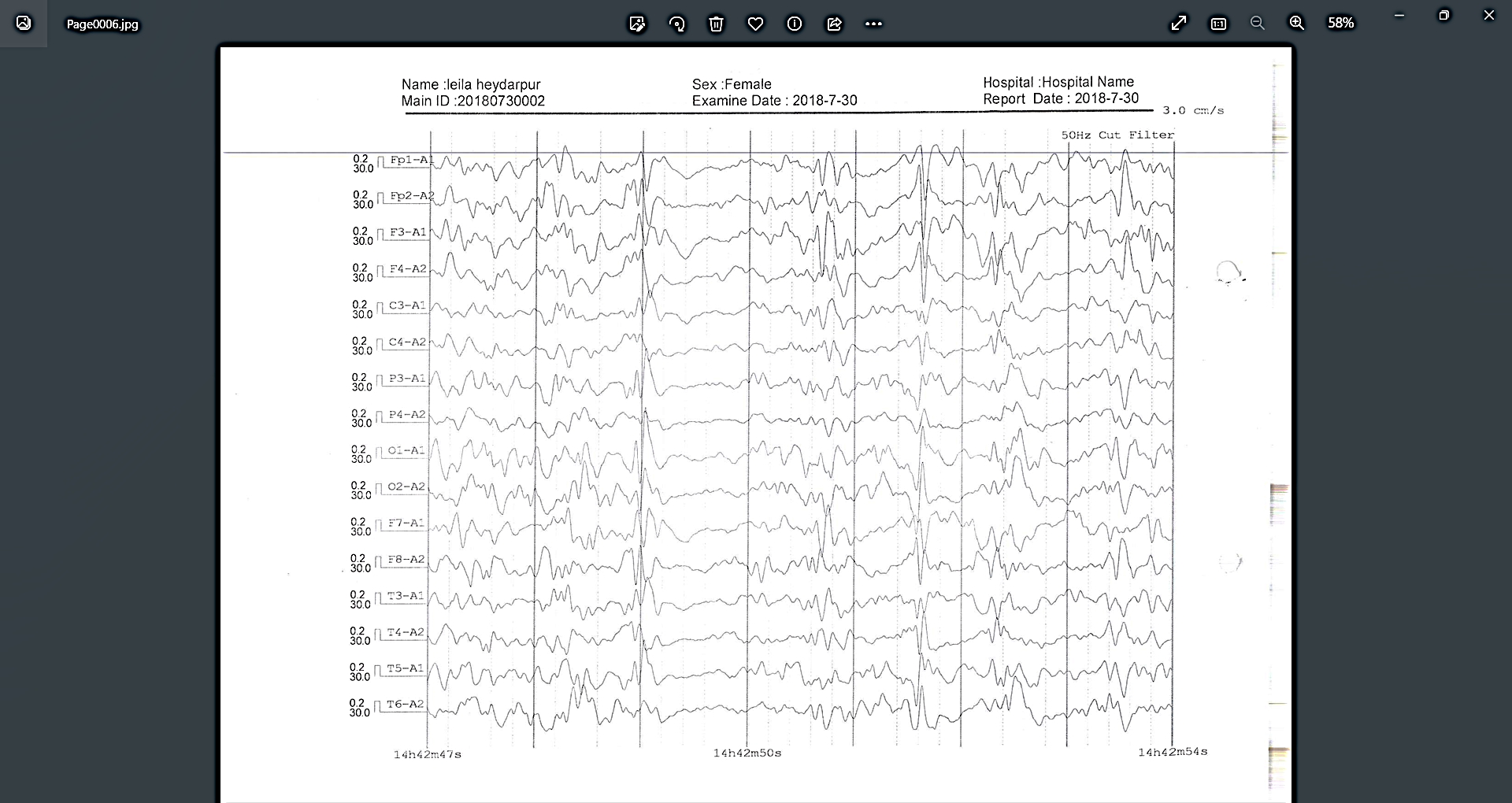


Interictal sleep EEG from patient II:3 (family 1) at 12 years showing a background of chaotic and disorganized activity with focal theta/delta bursts over the anterior regions and diffuse slow wave-sharp wave complexes. Note the absence of physiological sleep figures.

Interictal awake EEG from patient II:1 (family 3) at 13 years showing globally dysregulated background activity and asymmetric theta/delta bursts over the anterior and central areas and isolated slow wave-sharp wave complexes.


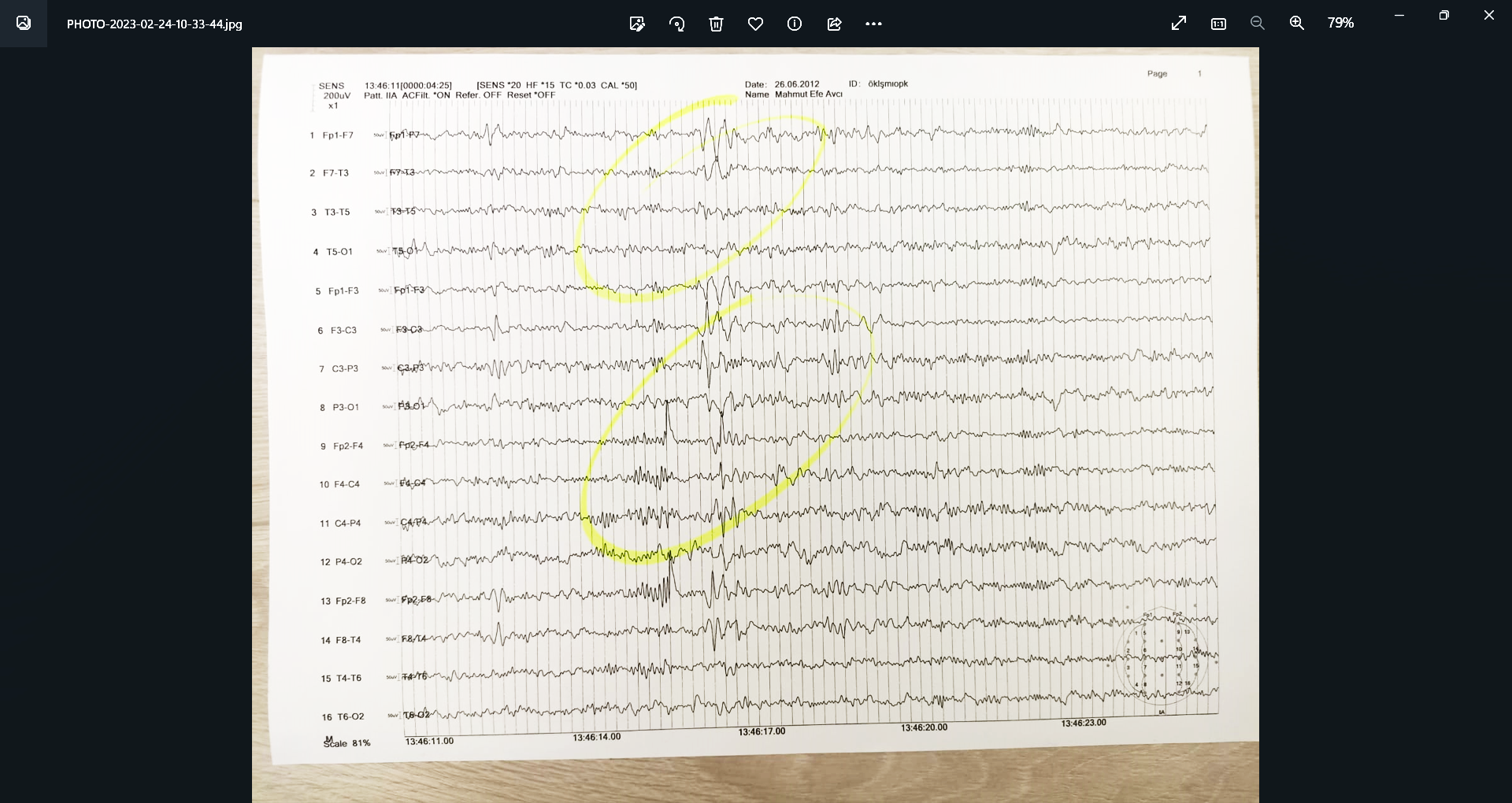


Interictal awake EEG from patient II:1 (family 4) at 2 years showing irregular background activity and bilateral spikes and slow wave discharges on the parasagittal region (period 2-3 sec. frequency 3-4 Hz) followed by suppressed baseline activity originated from bilateral central regions.


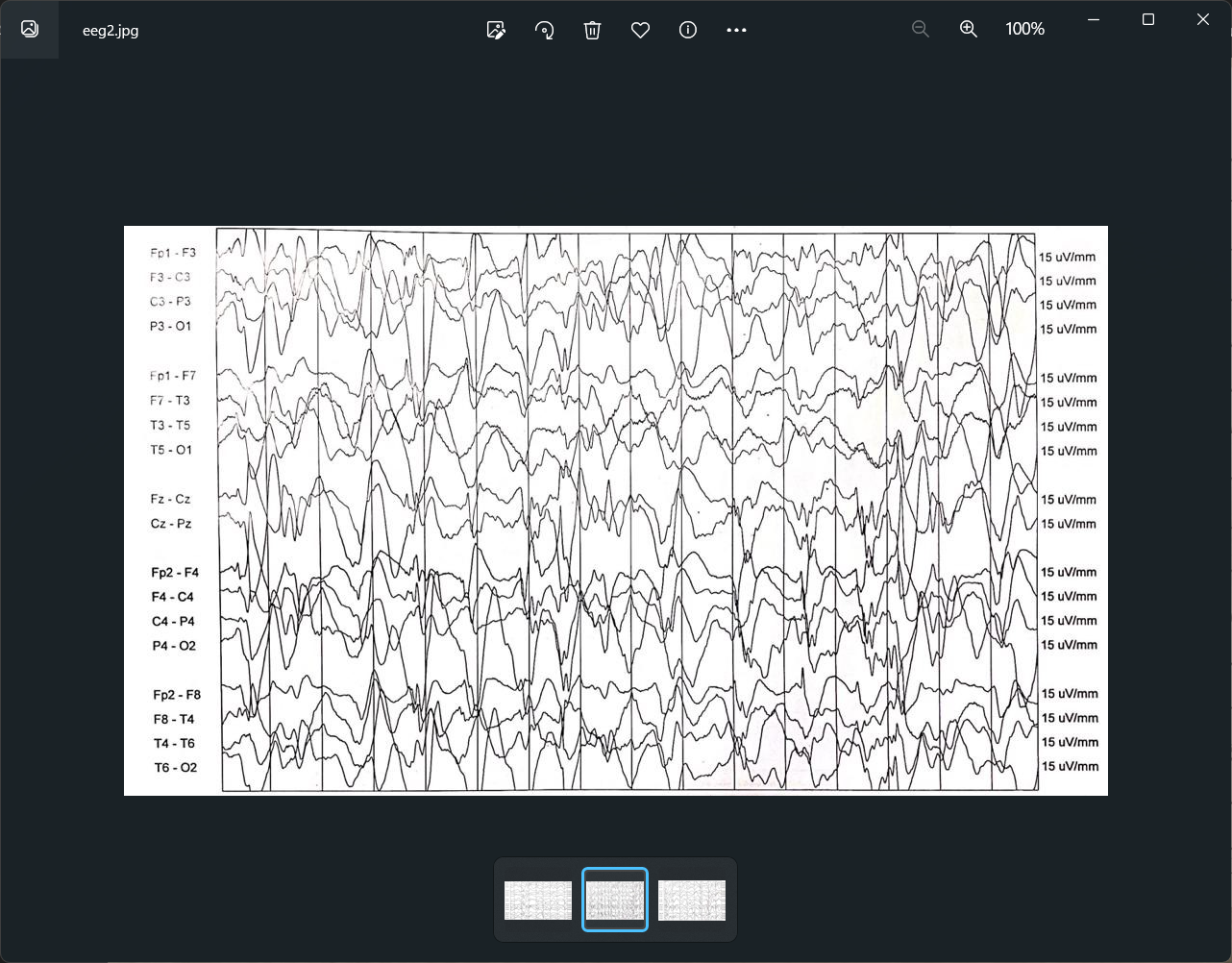


Interictal EEG from patient II:2 (family 5) at age 15 months showing slow-wave/sharp wave complexes within a background of chaotic and disorganized activity (modified hypsarrhythmia).

Interictal sleep EEG from patient II:2 (family 7)at 6 years shows generalized fast rhythms and disorganized background activity with a lack of normal sleep structure*.*
