# Supplementary material for: Lunapark deficiency leads to an autosomal recessive neurodevelopmental phenotype with a degenerative course, epilepsy and distinct brain anomalies
Source: Brain Commun. 2023 Aug 17;5(5):fcad222. doi: 10.1093/braincomms/fcad222 (PMC10546953; doi:10.1093/braincomms/fcad222)
Supplement: fcad222_Supplementary_Data [file fcad222_supplementary_data.zip › Supplementary Figure and Table Legends (JP).docx]

**Supplementary Figure 1** Brain MRI findings in all patients with *LNPK*-related disorder compared with a normal control individual. Sagittal T1 or T2-weighted (first), axial T2 or FLAIR (middle) and axial or coronal or sagittal T2 or FLAIR (last) images. All patients present hypoplasia of the corpus callosum (thick arrows) and the “ears-of-the-lynx” sign (thin arrows), variably associated with additional posterior periventricular white matter signal alterations (dotted arrows). In a subset of patients, white matter volume loss with a prevalent antero-posterior gradient and consequent ventricular dilatation and/or enlargement of the cerebrospinal fluid spaces was noted. A short midbrain is noted in ten patients (empty arrows).

**Supplementary Figure 2** Mild cerebellar atrophy in *LNPK*-related disorder. Brain MRI with axial and coronal T2-weighted images of patients II:3 of Family 1 (A, B), II:1 of Family 3 (C, D) and B-III-2 (E, F) (both performed at the age of 8 years) demonstrates mild enlargement of the cerebrospinal fluid spaces at the level of the lateral portions of the cerebellar hemispheres (empty arrows).

**Supplemental Table 1** Detailed genetic and phenotypic characteristics of patients with *LNPK* variants.

**Supplemental Table 2** Main features and comparison of disorders presenting with the ear-of-the-lynx sign.

**Supplemental material with EEG findings of patients with LNPK-related disorder**
